# SUMOylation Regulates Transcription by the Progesterone Receptor A Isoform in a Target Gene Selective Manner

**DOI:** 10.3390/diseases6010005

**Published:** 2018-01-02

**Authors:** Hany A. Abdel-Hafiz, Michelle L. Dudevoir, Daniel Perez, Mohamed Abdel-Hafiz, Kathryn B. Horwitz

**Affiliations:** 1 Department of Surgery, Anschutz Medical Campus, University of Colorado, 12801 E 17th Avenue, Aurora, CO 80045, USA; 2 Department of Medicine, Anschutz Medical Campus, University of Colorado, 12801 E 17th Avenue, Aurora, CO 80045, USA; michelle.dudevoir@ucdenver.edu (M.L.D.); Daniel.Perez@ucdenver.edu (D.P.); Kate.horwitz@ucdenver.edu (K.B.H.); 3 Department of Bioengineering, Anschutz Medical Campus, University of Colorado, 12801 E 17th Avenue, Aurora, CO 80045, USA; Mohamed.abdel-hafiz@ucdenver.edu; 4 Department of Pathology, Anschutz Medical Campus, University of Colorado, 12801 E 17th Avenue, Aurora, CO 80045, USA

**Keywords:** breast cancer, progesterone receptors, SUMOylation, tamoxifen resistance

## Abstract

Luminal breast cancers express estrogen (ER) and progesterone (PR) receptors, and respond to endocrine therapies. However, some ER+PR+ tumors display intrinsic or acquired resistance, possibly related to PR. Two PR isoforms, PR-A and PR-B, regulate distinct gene subsets that may differentially influence tumor fate. A high PR-A:PR-B ratio is associated with poor prognosis and tamoxifen resistance. We speculate that excessive PR-A marks tumors that will relapse early. Here we address mechanisms by which PR-A regulate transcription, focusing on SUMOylation. We use receptor mutants and synthetic promoter/reporters to show that SUMOylation deficiency or the deSUMOylase SENP1 enhance transcription by PR-A, independent of the receptors’ dimerization interface or DNA binding domain. De-SUMOylation exposes the agonist properties of the antiprogestin RU486. Thus, on synthetic promoters, SUMOylation functions as an independent brake on transcription by PR-A. What about PR-A SUMOylation of endogenous human breast cancer genes? To study these, we used gene expression profiling. Surprisingly, PR-A SUMOylation influences progestin target genes differentially, with some upregulated, others down-regulated, and others unaffected. Hormone-independent gene regulation is also PR-A SUMOylation dependent. Several SUMOylated genes were analyzed in clinical breast cancer database. In sum, we show that SUMOylation does not simply repress PR-A. Rather it regulates PR-A activity in a target selective manner including genes associated with poor prognosis, shortened survival, and metastasis.

## 1. Introduction

Actions of progesterone (P) are mediated by two progesterone receptor (PR) isoforms called PR-A and PR-B. They are co-expressed in the normal breast, but equimolarity may be disrupted in breast cancers [1]. The two isoforms have different physiological functions and regulate different sets of genes [1]. In animals, tissue-specific differences in their distribution patterns may account for dissimilar organ-specific P effects [2]. In the mouse mammary gland, PR-B is important for ductal side-branching and the labulo-alveolar development of pregnancy, while PR-A deficiency results in uterine and ovarian abnormalities and infertility. PR-A overexpression leads to abnormal mammary gland development including loss of cell–cell adhesion and basement-membrane integrity, suggesting that P signaling via PR-A contributes to tumorigenesis [3]. Some of the genes regulated only by PR-B and required for normal mammary gland development have been associated with metastasis [4].

In human cells, PR-A controls expression of genes involved in cell adhesion, cell morphology, invasiveness and metastasis, resistance to apoptosis, and tamoxifen resistance [5]. Overexpression of PR-A may promote tumorigenesis and modify responses to tamoxifen, but not to aromatase inhibitors [1]. In vitro and animal studies have shown that exogenous hormones can alter PR-A:PR-B ratios in both normal and malignant cells [6]. Similarly, exposure to exogenous hormones such as with HRT may alter PR-A:PR-B ratios in humans, including in breast cancers [1]. 

PRs are progestin-activated transcription factors that bind directly to DNA at progesterone response elements (PREs), or bind indirectly by tethering to other DNA-bound factors [7]. The functional differences between the two isoforms are not related to DNA binding since they have equal DNA binding affinities [8]. Rather, since the two isoforms have distinct structural conformations [9], they interact with dissimilar sets of co-regulators on DNA [10]. This is due in part to AF-3 in the unique PR-B upstream sequence and absent in PR-A, which contributes to PR-B’s strong transcriptional activity [11,12]. 

Post-translational modifications such as SUMOylation, acetylation, ubiquitination and phosphorylation modify all nuclear receptors thereby altering their hormone sensitivity, transcriptional activities, protein down-regulation, nuclear localization and protein-protein interactions [13]. Both PRs are post-translationally modified. PR N-termini contain most of the sites subject to modification, including the MAP kinase phosphorylation site Ser294, and the SUMO conjugation site Lys388. PR-B, but not PR-A, are phosphorylated on Ser294 by MAPK and cyclin dependent kinase 2 (CDK2) [14]. PR-B SUMOylation is highly dynamic and hormone-dependent. Disruption of Lys388 by mutation or by deSUMOylation increases the transcriptional activity of PR-B on synthetic PRE-driven promoters suggesting that SUMOylation inhibits transcription [15].

On PR-B, SUMOylation and phosphorylation are independent of one another [15]. However, activation of MAPK and its downstream kinases has complex effects on SUMOylation. For instance, expression of constitutively active MEKK1 has dual effects: it induces SUMOylation of unliganded PR-B but inhibits hormone-dependent PR-B SUMOylation [15]. At all concentrations, MEKK1 increases both basal and hormone-dependent PR-B transcriptional activity, suggesting that its effects on PR-B SUMOylation are indirect. Little is known about the role of SUMOylation on PR-A-dependent transcription of synthetic reporters; its effects on PR-A vs. PR-B function on model reporters; or its role in regulating PR-A vs. PR-B transcription on endogenous genes in breast cancer cells. In this study we address these questions.

## 2. Materials and Methods

### 2.1. Plasmids

Expression vectors for PR-B (pSG5 hPR1) and PR-A (pSG5 hPR2) were a gift of P. Chambon (Strasbourg, France). Cloning of pSG5 hPR1 K388R (PR-B K388R), pSG5 hPR2 K388R (PR-A K388R), hPR2 S294A (PR-AS294A), pSG5 hPR2 R606A (PR-A DX; a DNA dimerization mutant), and constitutively active pCMV-MEKK1 were described previously [7,11]. Construction of PRE_2_-Luc and MMTV-Luc reporter plasmids were described previously [12]. Flag-SENP1 and Flag-SENP1 mutant (R630L, K631M) were gifts of E. Yeh (M. D. Anderson, Houston, TX, USA).

### 2.2. Transcription Assays

HeLa cell transfections were carried out by calcium phosphate co-precipitation as described previously [11]. Briefly, 1.2 × 10^5^ cells were plated in phenol-red free minimum Eagle’s medium (MEM) containing 5% twice charcoal-stripped FBS. Twenty four hours later, cells were washed and treated with the synthetic progestin R5020 (Sigma Chemical Co., St. Louis, MO, USA), the mixed agonist/antagonist RU486, or the pure antiprogestin ZK98299 at the final concentrations indicated in the figures. Cells were harvested in lysis buffer (Promega, Madison, WI, USA), and the lysates were analyzed on a dual luminometer. Results were normalized to Renilla luciferase activity and expressed as indicated in the figures. 

### 2.3. Immunoblotting

Briefly, for Western blotting [16] whole cell extracts were prepared from transiently transfected HeLa cells treated as indicated in the figures. Following total protein quantification, lysates were separated by SDS-PAGE, transferred to nitrocellulose, and probed with anti-PR PgR1294 (DakoCytomation, Carpinteria, CA, USA) or anti-β-actin AC-74 (Sigma Chemical Co., St. Louis, MO, USA) monoclonal antibodies. The immunoreactive bands were visualized using the ELC system (PerkinElmer Life Sciences, Houston, TX, USA).

### 2.4. Stable Cell Lines

T47D human breast cancer cells stably expressing WT or SUMOylation-deficient PR-A were created by transfection of previously cloned PR negative T47D-Y cells with expression vectors that encode WT PR-A or PR-AK388R mutant [16,17]. Construction of PR-B expressing cells was previously reported. Cells were maintained in MEM supplemented with 5% FBS, nonessential amino acids, 25 U/mL penicillin and 25 μg/mL streptomycin, and 5 ng/mL insulin. All cell lines have been authenticated by short tandem repeat analysis and are routinely confirmed to be mycoplasma-free. 

### 2.5. Microarray Analysis

For gene expression profiling [18], PR-negative T47D-Y cells (no PR), plus derived cells stably expressing WT PR-A or the RPR-A K388 mutant, were cultured as above in vehicle control or 10 nM R5020 for 24 h. Total RNA was extracted (RNAeasy; Qiagen, Baltimore, MD, USA), cRNA was labeled and hybridized to U133 Plus 2 microarrays (Affymetrix, Santa Clara, CA, USA) as described [18]. Partek Genomics Suite 6.0 (Partek Inc., Palo Alto, CA, USA) was used to normalize raw data followed by ANOVA to identify genes differentially expressed among groups in a significant manner. A gene was considered significantly changed, if it had an adjusted *p* < 0.05 and fold-change ≥1.5. Heat maps and Venn diagrams were generated using Partek Suite 6.0. Ingenuity Pathway Analysis was used to identify biological processes enriched for differentially expressed genes. All microarray data have been deposited in the Gene Expression Omnibus Database (accession number GSE108607 (http//www.ncbi.nlm.nih.gov/geo)).

### 2.6. Gene Expression in Human Tumor Samples

The correlation between gene expression and survival in breast tumors was analyzed by Kaplan–Meier survival analysis (http://kmplot.com/analysis). We compared patient tumors that highly express the selected differentially regulated genes to ones with low expression and restricted the analysis to luminal subtypes that express ER and PR or were ER–/PR–. The hazard ratio with 95% confidence intervals and log rank *p*-value were computed. To carry out the comparative analysis between normal and tumor, we used the publicly available Oncomine database to find the status of differentially regulated genes in breast cancer vs. normal (www.oncomine.org) and used the differential analysis “cancer vs. normal” tool. The setting used for the searches were ATAD2, SPINT3, CDKN1c, and CLDN8; cancer type: Breast.

## 3. Results

### 3.1. SUMOylation Suppresses PR Transcriptional Activity in a Promoter Dependent Manner

**Figure 1:**
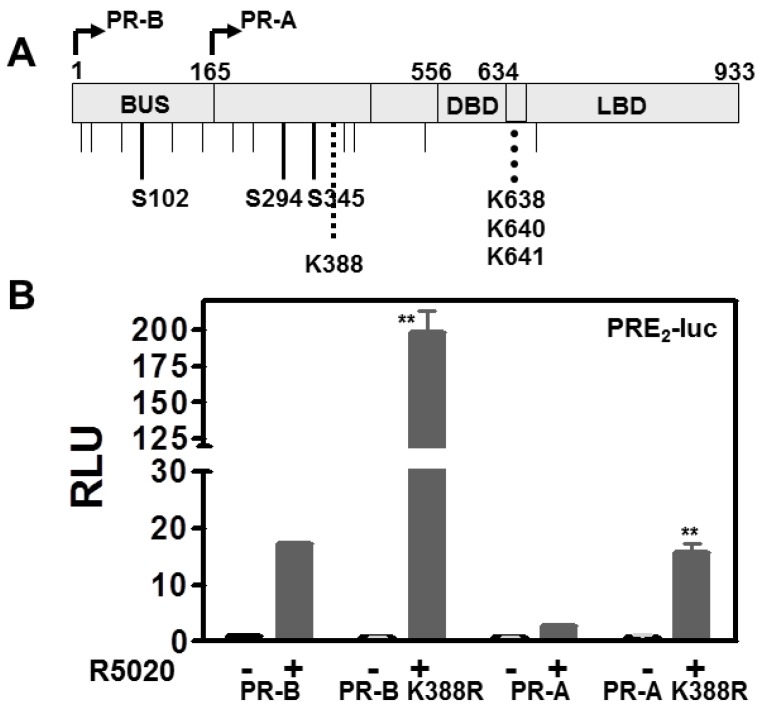

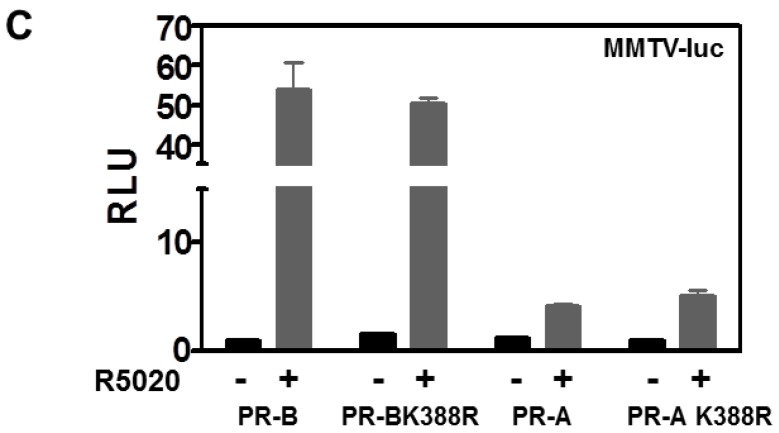
SUMOylation modulates PR transcriptional activity in promoter dependent manner. (**A**) Schematic representation of the progesterone receptors PR-A and PR-B showing the location of hormone dependent phosphorylation sites (S102, S294 and S345); SUMOylation site (K388); and acetylation site (amino acids 638–641). BUS, B-upstream segment; DBD, DNA binding domain; LBD, ligand binding domain. (**B**) HeLa cells were transiently transfected with PRE_2_-luciferase reporter, or (**C**) MMTV-Luc, together with 50 ng of WT PR-B, mutant PR-B K388R, PR-A, or mutant PR-A K388R expression vectors and Renilla-luciferase as described in Material and Methods. Transfected cells were treated for 24 h with 10 nM R5020. Luciferase activities are expressed in relative light units (RLU). Data represent triplicates (±SD). Statistical significance was computed by unpaired student’s *t* test. ** *p* < 0.05.

Downstream of aa165, PR-A and PR-B share post-translational modifications sites including ones for hormone-dependent phosphorylation (Ser294 and Ser345), SUMO conjugation (Lys388), and acetylation (KxKK at aa 638–641) (Figure 1A). PR-B SUMOylation is hormone-dependent and suppresses receptor-driven transcription on multiple PREs but not on a single PRE or on MMTV [15]. DeSUMOylation by mutation of PR-B Lys388 promotes transcriptional synergy with other factors on complex promoters. Since PR-A lacks the 165aa “B-upstream segment” (BUS) containing activation function 3 (AF-3), we conjectured that PR-A might not be subject to transcriptional synergy. We therefore compared effects of SUMOylation/deSUMOylation in HeLa cells transfected with WT PR-B or WT PR-A, and the PR-BK388R or PR-A K388R SUMOylation deficient mutants. All four were tested for ligand-independent (–) and ligand-dependent (+) transcription using PRE_2_-Luc (Figure 1B) and MMTV-Luc (Figure 1C) and the progestin R5020. There was no ligand-independent activity. In the presence of ligand, compared to their WT counterparts, both PR-B and PR-A SUMO-deficient mutants displayed increases in transcription on PRE_2_-luc (but not MMTV-Luc). Thus AF-3 of PR-B is not involved in de-repressing transcriptional activity mediated by SUMOylation. We confirmed that AF-3 was uninvolved using the PR-BdL140 BUS mutant in which we knocked out AF-3 function [12], by mutating its K388 to yield PR-Bdl140 K388R. This mutant nevertheless still demonstrated transcriptional upregulation, establishing on the full length PR-B molecule that AF-3 was unnecessary (data not shown). Note that while the activity of PR-A remains weak compared to PR-B it is nevertheless raised by deSUMOylation. Overall, based on use of synthetic promoters, this study demonstrates that SUMOylation suppresses transcription by PR-B and PR-A, which is relieved by deSUMOylation.

### 3.2. SENP1 deSUMOylates PR-A, Which Enhances Its Transcriptional Activity

**Figure 2:**
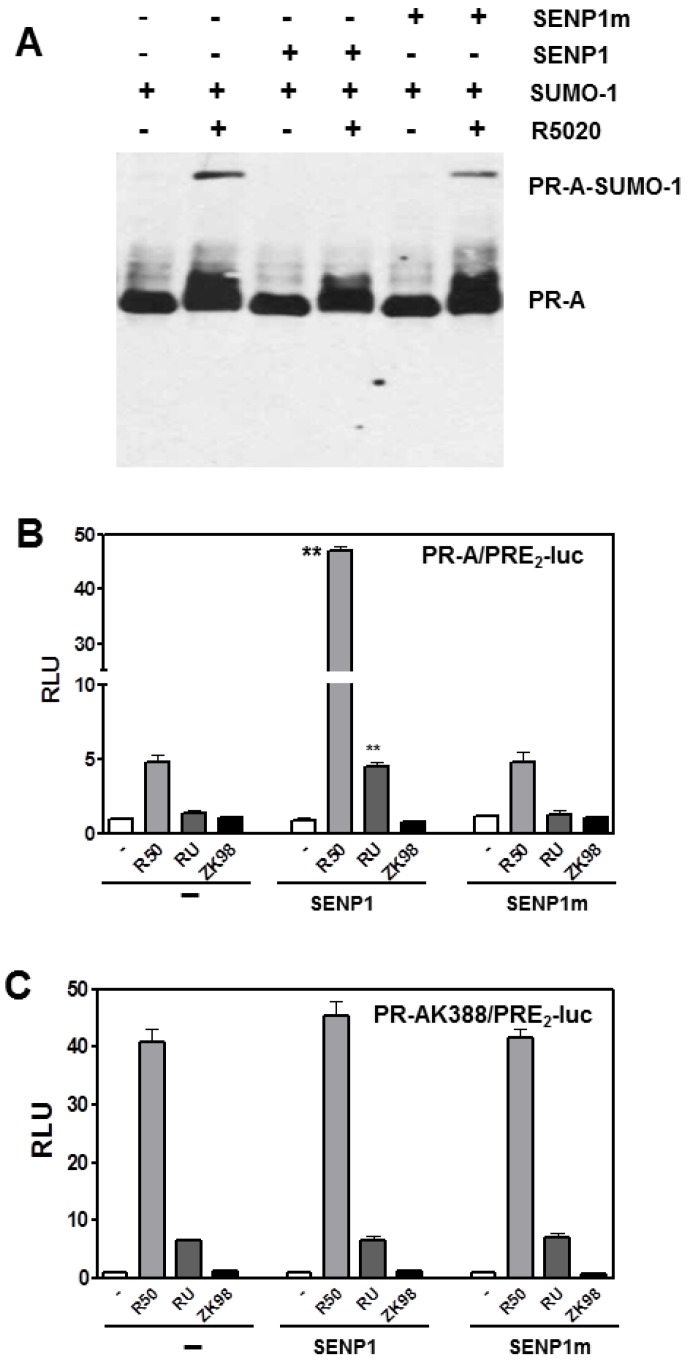
SENP1 deSUMOylates PR-A and enhances its transcriptional activity. (**A**) HeLa cells were transfected with GFP-SUMO-1, and WT or mutant (m) SENP1 vectors together with WT PR-A. Cells were treated with 10 nM R5020 for 24 h. Cells were lysed and analyzed by western blot for PR-A using anti-PR1294 monoclonal antibody. (**B**) HeLa cells were transiently transfected with WT PR-A or PR-A K388R, ** *p* < 0.05 (**C**) together with the PRE_2_-luciferase reporter plasmids and 100 ng of Flag-SENP1 or Flag-SENP1m mutant or an empty vector control (−). Transfected cells were treated with the agonist R5020 (R50-10 nM), the partial antagonist RU486 (RU-100 nM), or the pure antagonist ZK 98299 (ZK98-100 nM) for 24 h before being assayed for luciferase activity as described in Figure 1.

We analyzed SUMOylation in more detail focusing on PR-A. PR-B can be deSUMOylated by SENP1 [15]. Its catalytic function can be inactivated by mutation of Cys603 to yield mSENP1 [19]. We examined SENP1-mediated deSUMOylation of PR-A (Figure 2A) in HeLa cells co-transfected with PR-A, GFP-SUMO1, and either wild type SENP1 or mSENP1, in the absence or presence of R5020. To be SUMOylated, PR-A must be liganded (lanes 1, 3, 5). Liganded PR-A are SUMOylated in the absence of SENP1 (lane 2), remain SUMOylated with mSENP1 (lane 6) but are deSUMOylated by SENP1 (lane 4) demonstrating a role for SENP1 in regulating SUMOylation of PR-A. 

Based on Figure 1, this suggested that SENP1 would increase transcription by PR-A. To examine this, transcription of PRE_2_-Luc was measured in the presence of co-transfected SENP1 or mSENP1 when PR-A were liganded by the agonist R5020, by RU486 (a mixed agonist/antagonist on PR-B but a pure antagonist on PR-A), or by ZK98299, a pure antagonist on both PRs (Figure 2B). In HeLa cells expressing SENP1 or mSENP1, agonist-occupied PR-A are poor trans-activators and the antagonists have no effects. However, PR-A deSUMOylation by SENP1 increases R5020-dependent transcription 10-fold. Remarkably, deSUMOylation exposes the partial agonist properties of RU486 on PR-A; properties that had been reported previously only on PR-B [20]. Note that ZK98299 remains a pure antagonist even on deSUMOylated PR-A. Thus SUMOylation control the agonist/antagonist activity of RU486 liganded PR-A. Parenthetically, this suggests that PR-A may provide a sensitive assay for screening new antiprogestins for any partial agonist activities. In PR-A positive breast cancer cells, co-transfection of SENP1 but not mSENP1 also enhances transcription demonstrating that this effect is not cell specific (not shown). 

To confirm that the transcriptional effects in Figure 2B are due directly to PR-A deSUMOylation, a similar study was conducted using the SUMOylation deficient PR-AK388R mutant (Figure 2C). K388R mutant PR-A are completely insensitive to SENP1 demonstrating that the enzymatic effects in Figure 2B require an intact PR-A SUMOylation site. Interestingly, since either PR-A deSUMOylation or mutation of the SUMOylation site expose the partial agonist activity of RU486, both modifications may globally impact PR-A protein structure, thereby altering co-regulator recruitment.

### 3.3. The PR DNA Binding Domain (DBD) Dimerization Interface Is Unnecessary for SUMOylation or Transcriptional Control

**Figure 3:**
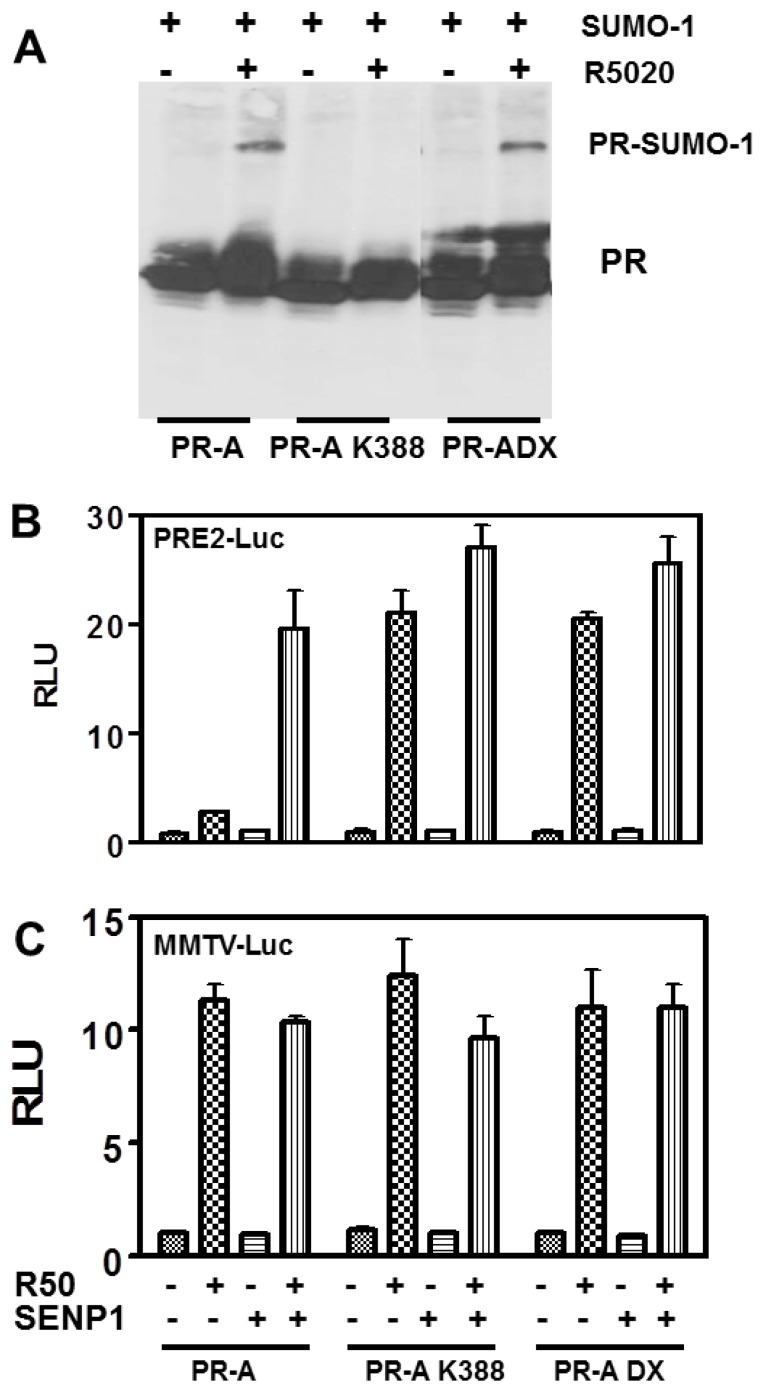
The PR DBD dimerization interface is not necessary for SUMOylation. (**A**) Western blot analyses were carried out as in Figure 2. HeLa cells were transiently transfected with GFP-SUMO-1, and expression vector encoding WT PR-A, PR-A K388R SUMOylation deficient mutant or DBD dimerization mutant PR-ADX (R606W). Cells were treated for 24 h with 10 nM R5020. The PR DBD dimerization interface is necessary for effective synergy control. HeLa cells were co-transfected with 2 μg of PRE_2_-luciferase (**B**), or MMTV-luciferase (**C**) reporters, together with 50 ng of a WT PR-A, the PR-A K388R SUMOylation deficient mutant, or a PR-A DBD dimerization mutant (PR-ADX) expression vectors and Renilla-luciferase as an internal control in the presence or absence of 100 ng SENP1 expression vectors. The cells were treated for 24 h with the agonist R5020 (10 nM), then harvested and lysed. The extracts were assayed for luciferase activities as in Figure 1.

Transcription by nuclear receptors is complex, involving post-translational modifications such as SUMOylation, a dimerization interface on the DBD [7,21], and for PR, interactions between the ligand binding domain (LBD) and N-termini to stabilize binding to DNA at multiple PREs [12]. To test the role of DBD dimerization, transcription was quantified on PRE2-luc using PR-A, the PR-A K388R mutant as control, and PR-A DX, an R606A mutant of the D-box in the second zinc finger of the DBD [7]. Figure 3A shows that unlike liganded PR-A K388R (lane 4), liganded PR-A DX (lane 6) are SUMOylated to levels equivalent to WT PR-A (lane 2). Thus, SUMOylation/deSUMOylation does not require an intact DBD or theoretically, DNA binding.

Transcription of PRE_2_ by WT and PR mutants is shown in Figure 3B. SENP1-mediated deSUMOylation strongly activates transcription by R5020-occupied WT PR-A. Mutation of K388 exposes this synergy even in the absence of SENP1. Similar hyper-activation is seen with PR-A DX whether or not it is deSUMOylated by SENP1. This shows that SUMOylation of the DBD dimerization mutant does not inhibit its activity at compound PREs, indicating that DBD dimerization is required downstream of SUMOylation to unmask the inhibitory effects of SUMO.

### 3.4. Stimulation of PR-A Ligand-Independent Transcription by MEKK Is Independent of SUMOylation

**Figure 4:**
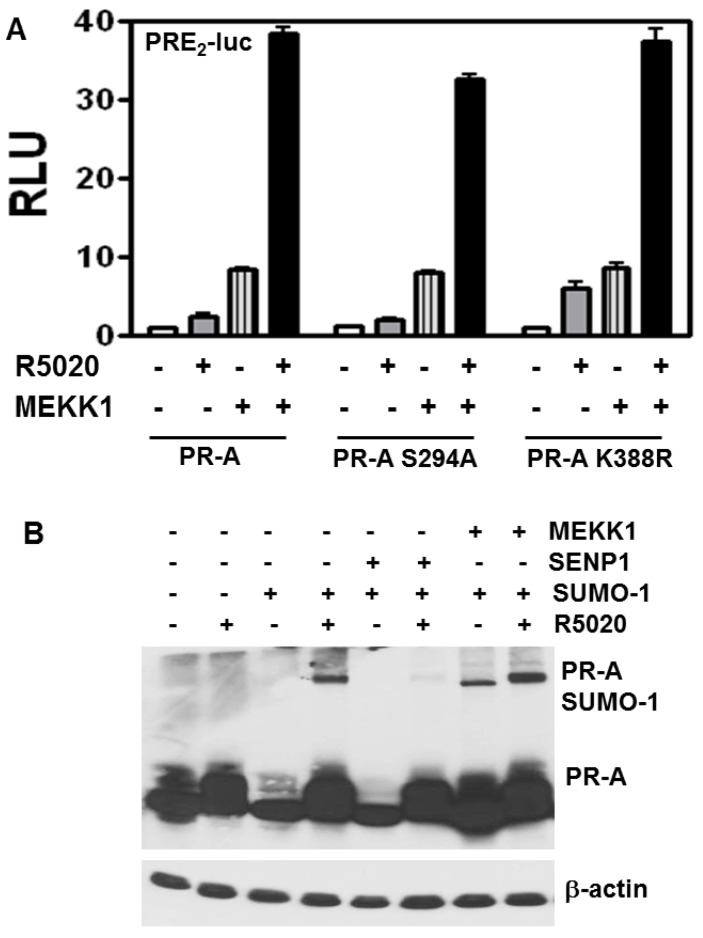
MAP kinase stimulatory effect on PR-A transcriptional activity is independent of the SUMOylation/deSUMOylation. (**A**) HeLa cells were transfected with 2 μg of PRE_2_-luciferase reporters together with 50 ng of WT PR-A, PR-A S294/345 phosphorylation mutant, or PR-A K388R SUMOylation deficient mutant expression vectors and Renilla-Luc as an internal control in the presence or absence of 100 ng constitutively active MEKK1 expression vector. The cells were treated for 24 h with the agonist R5020 (10 nM) then harvested and lysed. The extracts were assayed for luciferase activities as in Figure 1. (**B**) DeSUMOylation of PR-A by WT SENP1. HeLa cells were cot-ransfected with PR-A, GFP-SUMO-1 and SENP1 or MEKK1 expression vectors as indicated. Cells were grown in the presence (+) or absence (−) of R5020. PR in cell extracts separated on SDS-PAGE, were detected with anti-PR 1294 monoclonal antibody. β-actin served as a loading control.

To examine effects on transcription from the interplay between phosphorylation and SUMOylation, we compared transcription by WT PR-A, the K388R SUMOylation-deficient mutant, or a S294A phosphorylation-deficient mutant (Figure 4A) in the presence or absence of constitutively active MEKK1. Low MEKK1 expression levels increased hormone-independent and hormone-dependent activities of WT and K388R PR-A. Thus, the stimulatory effect of MEKK1 occurs even on deSUMOylated PR and thus independently of SUMOylation. MEKK1 also stimulated hormone-independent and hormone-dependent transcription of the S294A mutant, demonstrating that MEKK is not acting through this phosphorylation site. Of note, stimulatory effects of MEKK1 are highly concentration dependent. The data in Figure 4B use physiological MEKK1. At pharmacologic concentrations MEKK1 inhibits hormone-dependent PR-A SUMOylation (data not shown); underscoring the importance of dose-response studies in such analyses.

### 3.5. Histone Acetylation, SUMOylation and Transcription by PR-A

**Figure 5:**
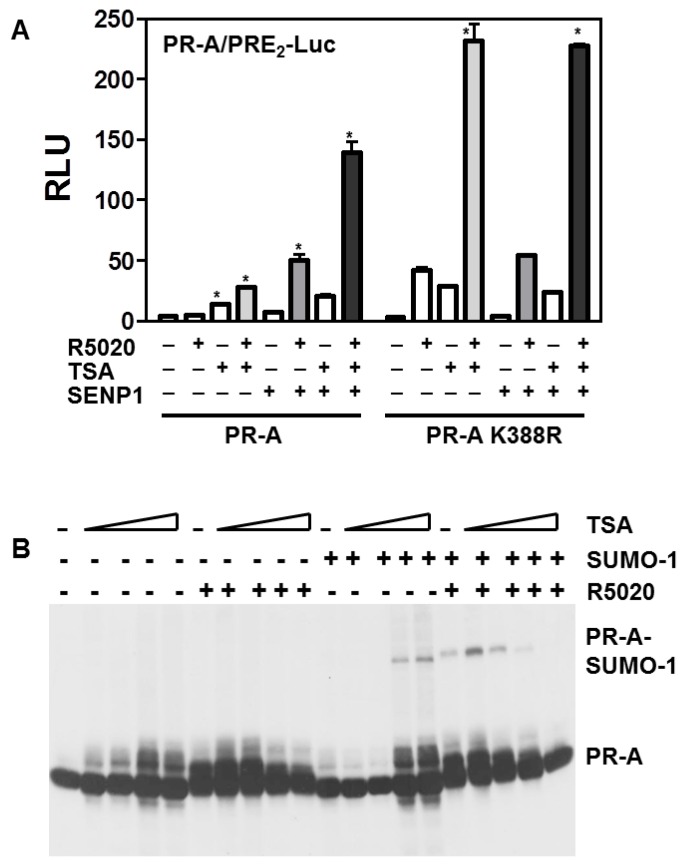
HDACs are not a major target for SENP1 action on PR transcriptional activity. (**A**) HeLa cells were transfected with 2 μg of PRE_2_-luciferase reporters together with 50 ng of a PR-A (**left**), or the PR-A K388R mutant (**right**) expression vectors and Renilla-Luc as an internal control in the presence or absence of 100 ng WT SENP1 expression vectors. The cells were treated for 24 h with the agonist R5020 (10 nM), without (−) or with (+) 100 nM TSA then harvested and lysed. The extracts were assayed for luciferase activities as in Figure 1. Statistical significance was computed by unpaired student’s *t* test * *p* < 0.05. (**B**) TSA induces ligand-independent PR-A SUMOylation. HeLa cells were transiently transfected with expression vectors encoding WT PR-A in the presence or absence of GFP-SUMO-1. Cells were treated 24 h without (−) or with (+) 10 nM R5020 in the presence of increasing concentration of TSA. Western blot analysis was performed on cell extracts probed with the anti-PR1294 monoclonal antibodies.

Trichostatin A (TSA) alters gene expression by impeding histone deacetylases (HDACs). HDACs have been implicated in transcriptional repression by SUMOylation [22]. To test whether HDACs influence SUMO regulation of PR, the transcriptional activities of WT PR-A or PR-A K388R were tested +/− TSA and +/− the SUMO de-conjugating enzyme SENP1 (Figure 5A). TSA alone improved transcription by unliganded or liganded PR-A, but to a lesser extent than deSUMOylation by SENP1 (left). TSA plus SENP1 were additive or weakly synergistic, suggesting relatively independent mechanisms of action for acetylation and SUMOylation. This was tested directly with the SUMOylation-deficient mutant (right). Here too, TSA boosted ligand-independent and -dependent transcription. Interestingly at high concentrations (Figure 5B), TSA induced SUMOylation of unliganded PR-A, but inhibited SUMOylation of liganded PR-A; a discordance which confirms that acetylation and SUMOylation are dissociated.

### 3.6. SUMOylation Differentially Regulates Endogenous Progestin Target Genes in Breast Cancer Cells

**Figure 6:**
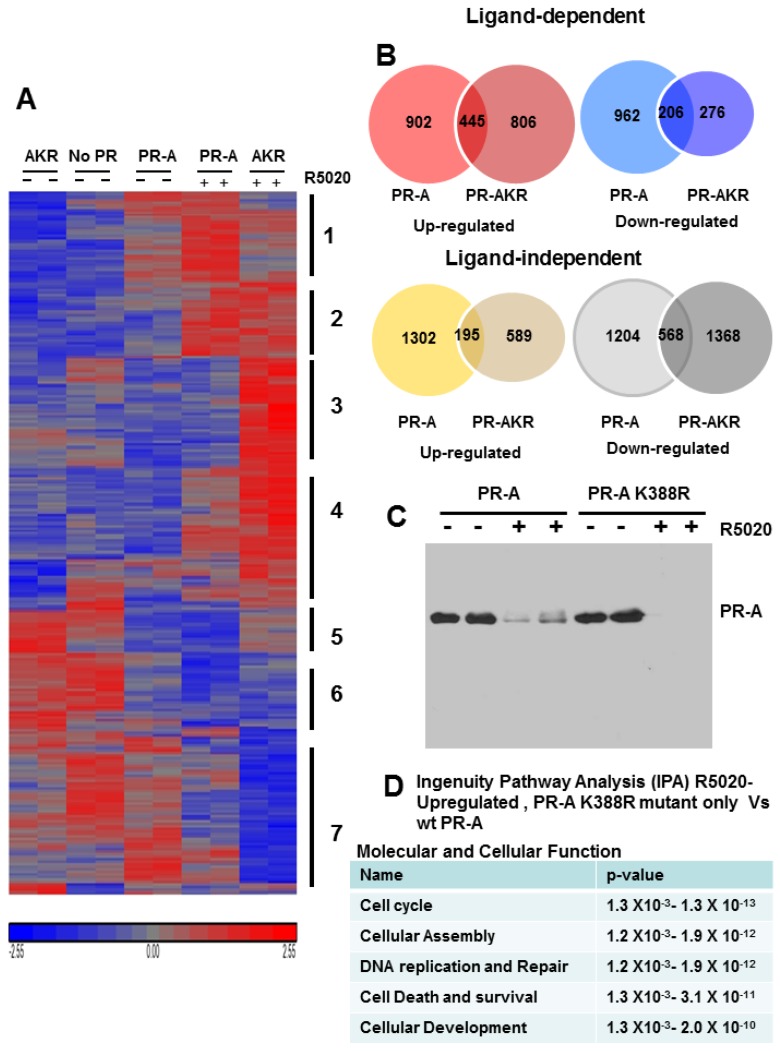
PR-A SUMOylation regulates the expression of endogenous genes. T47D stably transfected breast cancer cell lines with WT PR-A or PR-A K388R (AKR) SUMOylation deficient mutant were treated with 10 nM synthetic progestin R5020 (+) or vehicle for 24 h and isolated RNAs were analyzed by U133 Plus 2 microarray as described in “materials and methods”. (**A**) Heat map of R5020-regulated genes clustered by hierarchical clustering using Partek Genomics Suite 6.0. (**B**) Venn diagrams showing hormone-dependent up or down-regulated genes (**upper panel**) and hormone-independent up and down-regulated genes (**lower panel**). (**C**) Western blot shows the expression of WT PR-A or PR-A K388R SUMOylation deficient mutant in the presence (+) or absence (−) of 10 nM R5020. (**D**) Ingenuity pathway analysis (IPA) showing the top molecular and cellular functions that were the most significantly enriched for R5020-upregulated genes differently expressed between PR-A K388R mutant and WT PR-A cells.

The above synthetic promoter studies support the conclusion that SUMOylation exerts similar repressive effects on PR-A and PR-B. Can this conclusion be applied to endogenous genes? To answer this, we generated sublines from our previously generated PR-negative T47D-Y breast cancer line, that stably express WT PR-A or K388R PR-A (AKR). Figure 6C shows that T47D-Y are PR–; that the two modified cell lines express similar levels of PR in the absence of R5020; and that receptors in both cells undergo normal ligand-dependent down-regulation [16]. Further, both cell lines appropriately activate transcription of PRE_2_-Luc [16]. Transcript levels of the three untreated, or 24 h R5020-treated, cells were quantified using U133 Plus 2 chips [23]. Unliganded and progestin-regulated genes were grouped by hierarchical clustering into upregulated (red) and down-regulated (blue) genes (Figure 6A). Despite the complexity, statistical analyses resolved 7 distinct gene clusters (Figure 6A). In general Cluster 1 genes are upregulated uniquely by liganded WT PR-A; Cluster 2 genes are upregulated by WT and AKR mutant PR-A; Cluster 3 genes and to a lesser extent Class 4 genes are upregulated mainly by AKR mutant PR-A; Class 5 genes are interesting, apparently down-regulated by both unliganded and liganded WT PR-A; Class 6 genes tend to be down-regulated by liganded WT and mutant PR; Class 7 genes are demonstrably down-regulated mainly by the liganded AKR PR mutants.

Venn diagrams (Figure 6B) quantify progestin-regulated (red and blue) and ligand independent (yellow and grey) genes by WT PR-A or the SUMO-deficient AKR mutant. Four-hundred forty-five (445) genes were R5020-upregulated by both WT and mutant PR-A, 902 were upregulated only by WT PR-A, and 806 were upregulated only by the mutant. R5020-bound WT PR-A uniquely down-regulated 962 genes, the mutant down-regulated 276, and both receptors down-regulated 206. These diagrams clearly demonstrate the variable responses of endogenous genes to progestin-bound PR-A. The lower diagrams in Figure 2B quantify genes down- or upregulated by unliganded PR-A or PR-AK388R. These are of course also of considerable interest as they may modify tumor biology even in the absence of progesterone.

**Figure 7:**
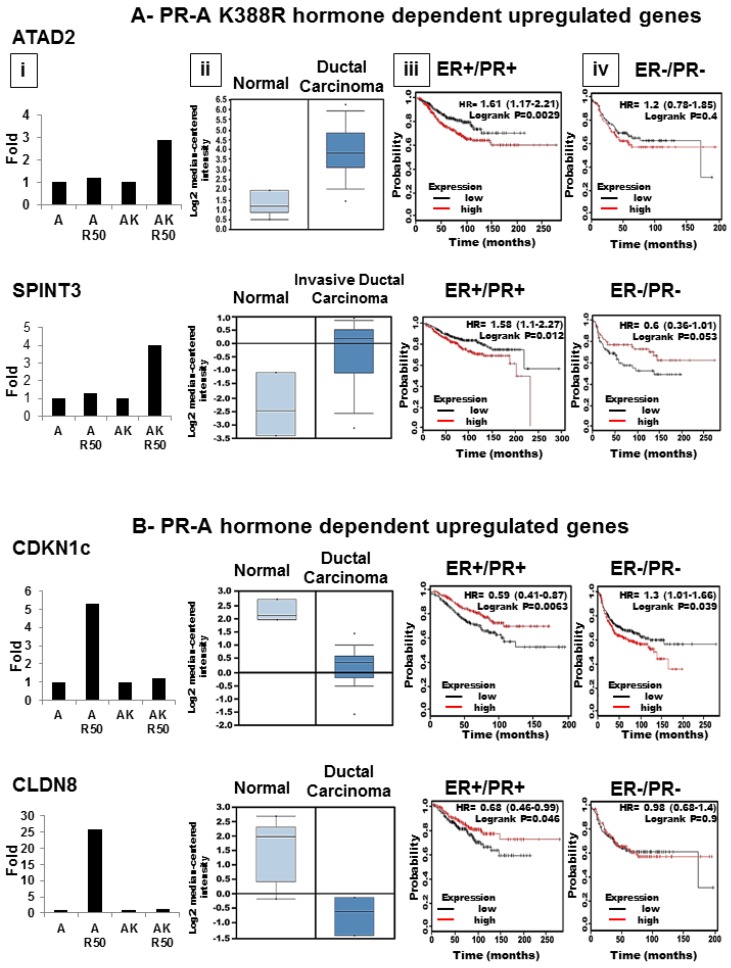
PR-A SUMOylation plays a functional role in tumor survival. Hormone dependent gene expression by PR-A K399R (**A**) or PR-A (**B**). Relative expression levels of ATAD2, SPINTS3, CDKN1c, and CLDN8 (PR-A target genes) in T47D breast cancer cells that stably express WT PR-A or PR-A K388R (**i**), and in breast cancer patient cohorts (**ii**) (Oncomine database). Kaplan–Meier survival curves showing relationship between high (red line) and low levels (black line) of target gene expression and overall survival in patients with ER-positive tumors (**iii**) or ER-negative tumors (**iv**). Statistical data are show in the upper right corner of each box with *p* ≤ 0.05 considered significant.

Expression profiling clearly shows that endogenously, SUMOylation modulates PR-A function in a target gene selective fashion, and not by general repression of PR-A activity. While some genes, such as SGK1 and PDK4 are unaffected by SUMOylation, others, such as CDKN1c and Claudin 8 (Figure 7) are only expressed in cells capable of SUMOylating WT PR-A. Expression of other genes, such as NDRG1, is enhanced only in the de-repressed K388R mutant cells, indicating that in WT cells SUMOylation represses NDRG1 activity. We used Ingenuity Pathway Analysis (IPA) to identify cellular functions and pathways significantly enriched by PR-A SUMOylation. The top five diseases were cancer, organismal injury and abnormalities, gastrointestinal disease, reproductive system disease and respiratory disease. The top five molecular and cellular functions were cell cycle, cellular assembly and organization, DNA replication, recombination and repair, cell death or survival, and cellular development (Figure 6D).

Several genes were selected using the Oncomine database, to assess their possible role in patients with breast cancer (Figure 7). ATAD2 and SPINT3 are upregulated by ligand in K388R mutant cells indicating that their expression is normally suppressed by SUMOylated PR (Figure 7(Ai)). Both genes are upregulated in ductal carcinomas (Figure 7(Aii)). Using Kaplan-Meier survival analysis (http://kmplot.com/analysis), we compared tumors that highly express these genes (red) to ones with low expression (black), in ER+/PR+ (Figure 7(Aiii)) or ER−/PR− disease (Figure 7(Aiv)). Significance data and *p*-values are shown in the upper right corner of each box. It is clear that upregulated genes may significantly (*p* ≤ 0.05) correlate with survival in some tumors. CDKN1c and Claudin 8 are strongly upregulated by progestin-occupied, SUMOylated WT PR-A. Receptor deSUMOylation suppresses this regulation. Interestingly, these genes are strikingly suppressed in ductal carcinomas and their expression may also correlate with tumor behavior. In general, we hypothesize that besides acting as markers, PRs and their SUMOylation/deSUMOylation state may play a functional role in tumor survival.

## 4. Discussion

ER+/PR+ tumors respond better to endocrine therapies than ER+/PR– ones but PR levels are not necessarily related to benefit, raising questions about appropriate PR “cut-off” levels in clinical assays. Additionally, besides simply acting as markers of hormone responsiveness, PR signaling undoubtedly has functional consequences in both liganded and unliganded states. The receptors are extensively modified post-translationally, which influences their gene regulatory capacity including crosstalk with ER [24,25]. For instance it is not always appreciated that PR can inhibit ER transcriptional activity [11,24].

Moreover, all P target tissues express two PR isoforms, which regulate different sets of genes with different downstream sequelae [4]. PR-B but not PR-A are required for mammary gland development and expansion [26]. RANKL, a gene activated only by PR-B [27], plays an important role in the pregnant mammary gland; in initiation and progression of P-induced breast cancer [28]; and for maintenance and expansion of mammary stem cells [29]. The structural and functional mechanisms underlying differences between PR-A and PR-B are under active investigation. With respect to structure, we find that we can completely destroy the AF-3 transcriptional function unique to the N-terminal region of PR-B, without converting the mutant to PR-A. Differences in function are unrelated to DNA binding affinity since both receptors bind DNA equally well. Rather, it appears that functional differences are dependent on recruitment of different coactivators to the two DNA-bound receptors [30]. This recruitment in turn is controlled by post-translational modifications of the receptors, including their phosphorylation, ubiquitination, acetylation and SUMOylation states. Here we focus on SUMOylation of PR-A, and examine phosphorylation and acetylation, to analyze the consequences of these modifications on transcription of synthetic promoter/reporters, and also on transcription of endogenous genes in human breast cancer cells. The latter have rarely been assessed.

We use PR-negative human breast cancer cells that we isolated, then modified to stably express WT PR-A, a SUMOylation deficient PR-A K388R mutant to model SUMOylation failure, plus other mutants. We also use various concentrations of enzymes that de-SUMOylate the receptors or drugs that target phosphorylation or acetylation. With regard to SUMOylation, our analyses indicate that this modification does not simply attenuate PR activity on all target genes as has been concluded from use of synthetic promoters. Instead endogenously, PR SUMOylation controls whether the receptors either liganded or unliganded, up- or down-regulate genes, or lose regulatory capacity altogether. This endogenous complexity is consistent with previous reports for PR-B, and for glucocorticoid (GR) and androgen (AR) receptors, which also show SUMO-sensitive target-selectivity [31]. 

We report pathway analysis to identify genes differentially regulated by SUMOylated vs. deSUMOylated PR-A, and find that the dominant genes are involved in molecular and cellular functions of the cell cycle, cellular assembly and organization, DNA replication, recombination and repair, cell death/survival, and cellular development. The top genes upregulated by liganded but deSUMOylated PR-A (i.e., genes that are normally repressed by receptor SUMOylation) are associated with brain metastasis, poor overall survival and poor prognosis. Among these are: ATAD2, ADAMTS8, DIO2, KCNMA, and NOK1. Briefly, ATAD2 is a nuclear coactivator of ER and AR crucial for assembly of chromatin-modifying complexes and proliferation of hormone-responsive cancer cells [32]. Its overexpression correlates with aggressiveness of breast and endometrial cancers [33] via recruitment of the coactivator SRC3 [34]. ADAMTS8 is also a predictor of poor overall survival [35]. DIO2 activates thyroid hormone and plays a role in P/PR-induced proliferation [36]. KCNMA1 is a pore forming α-subunit of the large-conductance calcium- and voltage-activated potassium channel linked to heightened proliferation and brain metastases [37]. NOK is a potent oncogene expressed in multiple cancers [38], which activates signaling pathways including MAPK and PI3K by phosphorylating them [39]. Clearly, all these genes are important and subject to complex regulation, among which are the “repressor” effects of SUMOylated PR-A.

Then, we find genes that require liganded SUMOylated PR-A for their upregulation. One is *CDKN1C*-p57Kip2 (Figure 7), a protein reduced or lost in the majority of breast cancers, possibly through ER-dependent mechanisms. Similarly, Claudin 8 (CLDN8), a tight junction protein dysregulated in a number of cancers [40], is down-regulated in ductal carcinomas compared to the normal breast (Figure 7) and is associated with differential survival of patients with ER+/PR+ vs. ER−/PR− disease. Another interesting gene in this category is C/EBPδ, an inflammatory response gene and tumor suppressor associated with metastasis [41]. Other genes uniquely upregulated by SUMOylated PR-A include ALDH1A3 (4.5-fold), Annexin A1 (5.6-fold) and ERRγ, an estrogen-related receptor induced 26.2-fold. All are associated with poor patient survival, triple negative disease and tamoxifen resistance, and all are examples of genes that are not suppressed by PR-A SUMOylation.

In sum, our studies address PR structural regions and signaling pathways involved in post-translational PR-A modifications, focused on transcriptional regulation by SUMOylation. We demonstrate that the mixed progestin/antiprogestin RU486, used to terminate early pregnancy by blocking P-activated PRs, loses its antagonist properties on de-SUMOylated receptors. This is an interesting example of how a receptor’s SUMOylation state modifies the target specificity of a bound hormone. Parenthetically, this suggests use of SUMO-deficient PR-A as a sensitive assay for screening new antiprogestins for their partial agonist activities. Only a pure antiprogestin would be inactive on de-SUMOylated PR-A. We also show that the PR-A SUMOylation state controls not only the transcriptional potency of these receptors, but also the direction, both up and down, in which endogenous genes are progestin-regulated through PR-A. These issues need to be understood, especially in premenopausal women, as SUMO-modifying drugs enter mainstream medical practice for treatment of cancers, cardiac disease, neurodegenerative diseases, viral infections, and the like [42].
